# Heterozygous frameshift mutation in *FaMYB10* is responsible for the natural formation of red and white-fleshed strawberry (*Fragaria* x *ananassa* Duch)

**DOI:** 10.3389/fpls.2022.1027567

**Published:** 2022-10-26

**Authors:** Huazhao Yuan, Weijian Cai, Xiaodong Chen, Fuhua Pang, Jing Wang, Mizhen Zhao

**Affiliations:** Institute of Pomology, Jiangsu Academy of Agricultural Sciences/Jiangsu Key Laboratory for Horticultural Crop Genetic Improvement, Nanjing, China

**Keywords:** *Fragaria x ananassa*, flesh color, anthocyanin, *FaMYB10^AG-insert^*, heterozygous

## Abstract

During natural evolution and artificial selection, the fruit color of many species has been repeatedly gained or lost and is generally associated with mutations in genes encoding *R2R3-MYB* transcription factors, especially *MYB10*. In this study, we show that a heterozygous frameshift mutation (*FaMYB10^AG-insert^/FaMYB10^wild^*) is responsible for the loss of anthocyanins in the flesh of cultivated strawberry. Comparative transcriptomic and metabolomic analyses of red- and white-fleshed strawberry indicated that the low expression level of *FaUFGT* (flavonol-O-glucosyltransferases) was responsible for the loss of anthocyanins and accumulation of proanthocyanidin in the white-fleshed strawberry and was the crucial gene that encodes enzymes of the anthocyanin biosynthesis pathway. Accordingly, overexpression and silencing of *FaUFGT* altered anthocyanin content and changed the flesh color of strawberry fruits. Furthermore, whole-genome resequencing analyses identified an AG insertion in the *FaMYB10* coding region (*FaMYB10^AG-insert^*) of white-fleshed strawberry. Y1H and EMSA assays showed that FaMYB10^wild^ was able to bind to the promoter of the *FaUFGT* gene, while the FaMYB10^AG-insert^ could not. The skin and flesh color were tightly linked to the number of fully functional *FaMYB10* copies in the selfing progeny of white-fleshed strawberry. Our results suggested that heterozygous frameshift mutation of *FaMYB10* resulted in the loss of the ability to activate the expression of the *FaUFGT* gene, was responsible for the natural formation of red and white-fleshed strawberry.

## Introduction

Cultivated strawberry (*Fragaria* x *ananassa* Duch.) is a horticultural crop cultivated worldwide that has considerable economic value because of its strong aroma, rich flavor, attractive appearance and abundant nutritional value (Yuan et al., 2019). It originated from the accidental crossing between two wild octoploid species, white-fruited *Fragaria chiloensis* and red-fruited *Fragaria virginiana*, in France nearly 300 years ago (Finn and Ramos, 2013). The number and diversity of metabolites give rise to these desirable horticultural traits in strawberry fruits, especially bright fruit color. The quantity and variability of flavonoids control strawberry fruit coloration in cultivated octoploid strawberry varieties (Wang et al., 2019). Flavonoids are ubiquitous secondary metabolites that possess a variety of biological activities, such as defense against ultraviolet rays and biological and abiotic stresses (Harborne and Williams, 2000; Treutter, 2005; Nenaah, 2014). Additionally, flavonoids are believed to be good for human health, serving such functions as preventing cardiovascular diseases and controlling obesity and antitumoral effects (Lin et al., 2016). Branches of the flavonoid biosynthetic pathway are involved in the production and regulation of anthocyanins, proanthocyanins and flavonols. Anthocyanins are water-soluble flavonoids that are responsible for fruit color (Tanaka et al., 2008). Pelargonidin-3-glucoside and cyanidin-3-glucoside are considered to be the most common anthocyanin pigments accounting for strawberry fruit color (Da Silva et al., 2007; Giampieri et al., 2012).

The anthocyanin biosynthetic pathway has been rigorously studied and is associated with a number of crucial genes that encode enzymes of the anthocyanin biosynthesis pathway (Fischer et al., 2014; Zhang et al., 2015; Hawkins et al., 2017). Manipulating any step of the expression of these genes can alter anthocyanin content and the subsequent color of plants. For example, antisense inhibition of the *F3H* gene led the original orange-red flower color to become light or even colorless in carnation (Zuker et al., 2002). In the last step, UFGT attaches sugar molecules to anthocyanidins to increase their stability. Silencing the gene encoding UFGT reduces the anthocyanin content but induces the proanthocyanidin content in strawberry fruits (Griesser et al., 2008).

It is well known that the expression of genes that encode enzymes of the anthocyanin biosynthesis pathway is primarily regulated by a conserved ternary MYB-bHLH-WD40 (MBW) complex in the anthocyanin biosynthetic pathway, which consists of the R2R3-MYB, bHLH and WD40 proteins (Schaart et al., 2013; Wang et al., 2013). R2R3-MYB gene family members have been described as master regulators in various fruit species, such as strawberry (Wang et al., 2010), apple (Espley et al., 2007), pear (Feng et al., 2010), citrus (Huang et al., 2020), peach (Rahim et al., 2014) and sweet cherry (Jin et al., 2016). In the process of natural evolution and artificial selection, the fruit color of many species is repeatedly gained or lost, which generally involves mutations in the *R2R3-MYB* transcription factor. The natural variation in anthocyanin content in most citrus species is caused by multiple mutational patterns of the *Ruby1* gene, which are predicted to be nonfunctional for anthocyanin accumulation (Butelli et al., 2017). *Ruby2* is adjacent to *Ruby1* in the citrus genome and has the same function in the regulation of anthocyanin accumulation. Notably, two alleles, *Ruby2^Short^* and *Ruby2^Full^*, exist in pummelo and orange, respectively, which have opposite functions in the regulation of anthocyanin accumulation (Huang et al., 2018). Similarly, mutation of the *MYB* transcription factor leads to changes in fruit color in the course of *Fragaria* evolution (Wang et al., 2019; Castillejo et al., 2020).

Many different types of *MYB10* loss-of-function mutations exist in *F. vesca* species that result in white-fruit phenotypes. For instance, a gypsy retrotransposon insertion in *MYB10* results in a truncated protein and the loss of the regulatory function of anthocyanin biosynthesis (Castillejo et al., 2020). An ACTTATAC insertion in the *FaMYB10* coding sequence of the white cultivated strawberry (*Fragaria* x *ananassa*) ‘Snow Princess’ results in a predicted truncated protein, which leads to the loss of function of binding to the bHLH and WD40 partner proteins and regulates anthocyanin biosynthesis (Wang et al., 2019). Natural variation in the *MYB10* transcription factor promoter also affects anthocyanin accumulation in fruit. A CACTA-like transposon (FaEnSpm-2) insertion in the *FaMYB10* promoter of red-fleshed strawberry varieties enhances anthocyanin synthesis gene expression and anthocyanin accumulation (Castillejo et al., 2020). Inactivation of the *FnMYB10* promoter caused by mutation in the core element region leads to low expression of the *FnMYB10* gene, which is related to the white color of *Fragaria nilgerrensis* fruit (Zhang et al., 2020).

In addition to the *MYB10* gene, many other *R2R3-MYB* gene family members, such as *FaMYB1* (Paolocci et al., 2011), *FaMYB5* and *FaMYB9/FaMYB11* (Schaart et al., 2013), have been determined to be involved in the regulation of anthocyanin accumulation in cultivated strawberry (Lin-Wang et al., 2010; Kadomura-Ishikawa et al., 2015). The products of these genes interact with the bHLH transcription factor or WD40 protein to form an MBW complex, which activates or inhibits anthocyanin biosynthetic pathway genes. Mutation in the coding region of *FaMYB10* has been associated with the white fruit phenotype of ‘Snow Princess’ (Wang et al., 2020), but exactly how it regulates genes involved in anthocyanin biosynthesis is unclear. The gene regulatory network of the anthocyanin biosynthetic pathway has not been fully elucidated in octaploid-cultivated strawberry. In addition, all of the genes in this pathway have been studied in whole strawberry fruit, which does not distinguish between skin and flesh.

In this study, comparative transcriptome, metabolome, and whole-genome resequencing analyses were performed to study the flesh coloration mechanism using two cultivated strawberry varieties, specifically ‘Benihoppe’ (HJ) with red skin and flesh and ‘Xiaobai’ (XB) with white flesh and red skin. In addition, *FaUFGT* was also transiently overexpressed in HJ and XB fruit. Furthermore, Y1H and EMSA assays was used to verify if FaMYB10^wild^ and FaMYB10^AG^ could be able to bind to the promoter of the *FaUFGT* gene. We also analyzed the correlation of *FaMYB* genotype with skin color and flesh color in the selfing progeny of XB strawberry. These results provided new details to further the understanding of the mechanism of color formation in red- and white-fleshed strawberry.

## Results

### Analyses of anthocyanin components in strawberry flesh and skin

**Figure 1:**
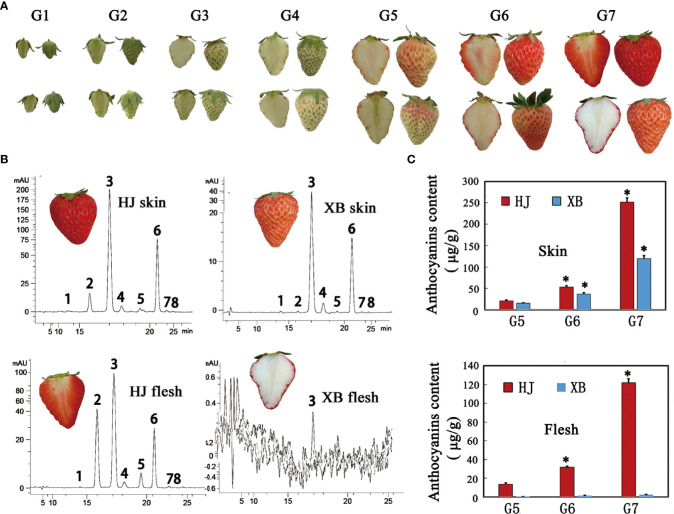
Phenotypes and anthocyanin content of flesh and skin during strawberry fruit ripening. **(A)** Images showing the flesh and skin of HJ and XB during strawberry fruit ripening. **(B)** HPLC chromatograms at 520 nm for anthocyanins in the flesh and skin of strawberry ripening fruit cv HJ, cv XB, respectively. The four panels represent anthocyanins in HJ skin, HJ flesh, XB skin, and XB flesh according to top-to-bottom and left-to-right scanning. Eight peaks from left to right: cyanidin-3,5-di-O-glucoside, cyanidin-3-glucoside, pelargonidin-3-glucoside, peonidin-3-glucoside, cyaniding-3-O-(6-O-malonyl-b-D-glucoside), pelargonidin-3-O-(6-O-malonyl-b-D-glucoside), pelargonidin-3-O-methyl-(6-O-malonyl-b-D-glucoside) and peonidin-3-O-methyl-(6-O-malonyl-b-D-glucoside). **(C)** The total anthocyanin contents of flesh and skin during strawberry fruit ripening. Three replications were performed, and the error bars represent ± SE. Significant differences were determined using independent t-tests. "*" represented p-value < 0.05.

HJ is a commercial strawberry variety in China that has red skin and flesh. XB has white flesh and red skin, which are obtained by tissue culture mutation. There was a significant difference in skin and flesh coloration between HJ and XB (**Figure 1A**). We divided the development of strawberry fruit into seven developmental stages: small green (G1), large green (G2), degreen (G3), white (G4), initial red (G5), partial red (G6), and full red (G7), representing a minor adjustment in the developmental stages reported in previous studies (Jia et al., 2011; Yuan et al., 2019). Eight anthocyanin components were detected in the skin and flesh of HJ and XB ripening fruit by HPLC-MS/MS (**Supplementary Information 1**: **Table S1**). Pelargonidin-3-glucoside was the major anthocyanin substance (**Figure 1B**). Overall, there was a higher anthocyanin content in the skin than in the flesh (**Figure 1C**). Anthocyanin was detected in the skin of XB during the G5, G6 and G7 developmental stages, but its content was considerably lower than that in the skin of HJ. Consistent with the white color phenotype, the contents of anthocyanin were almost undetectable in the flesh of XB. In contrast, there was a high anthocyanin content in the flesh of HJ during the G5, G6 and G7 developmental stages.

### Comparison of the expression patterns of genes involved in anthocyanin biosynthesis between red (HJ) and white (XB) strawberry flesh

**Figure 2:**
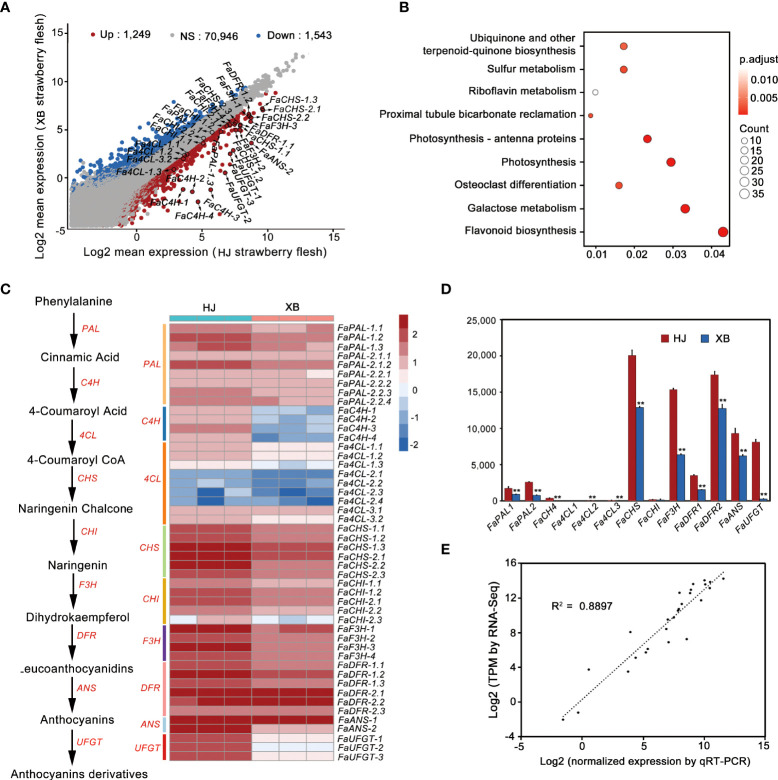
Comparison of the expression patterns of genes participating in anthocyanin biosynthesis between red (HJ) and white (XB) strawberry flesh. **(A)** Analysis of the differentially expressed genes in HJ and XB ripe flesh detected by RNA-Seq. **(B)** KEGG analysis of the Differentially expressed genes in XB vs. HJ. **(C)** The expression pattern of genes participating in anthocyanin biosynthesis in HJ and XB ripe flesh detected by RNA-Seq. Enzyme names, corresponding transcripts and expression patterns were located at the side of each step. Heat map showing log_10_ (TPM) values of expression for each transcript in ripe HJ and XB flesh. **(D)** The expression pattern of genes participating in anthocyanin biosynthesis in ripe HJ and XB flesh detected by RT-qPCR. **(E)** Correlation of the expression level obtained from RNA-Seq and RT-qPCR. Three replications were performed, and the error bars represent ± SE. Significant differences were determined using independent t-tests. "**" represented p-value < 0.01.

To explore the candidate genes controlling strawberry flesh color formation, we also performed a whole transcriptome sequencing of HJ and XBflesh at full red (G7) stage. Among the mRNA transcripts, 2792 differentially expressed genes (DEGs) were found in XB vs. HJ, with 1,249 being up-regulated and 1,543 being down-regulated (**Figure 2A**, **Supplementary Information 2**). KEGG analysis revealed that DEGs were most enriched in flavonoid pathways (**Figure 2B**). Forty-eight genes belong to flavonoid biosynthesis pathway in strawberry (*Fragaria* x *ananassa* Duch.) genome, and more than 60% (30 genes) of them being down-regulated gene (**Figures 2A, C**). The majority of transcripts participating in anthocyanin biosynthesis were slightly more highly expressed in ripe HJ flesh than in ripe XB flesh, regardless of whether early genes (*FaPAL*, *FaCHS*, *FaCHI*, and *FaF3H*) or late genes (*FaDFR* and *FaANS*) were included (**Figures 2A, C**, and **Supplementary Information 3**). However, *FaUFGT*, the last key gene in the anthocyanin biosynthesis pathway, showed significantly higher expression (more than twenty times higher) in ripe HJ flesh than in ripe XB flesh. Compared with anthocyanin biosynthesis genes (*FaCHS*, *FaF3H, FaDFR*, *FaANS* and *FaUFGT*), the key genes in the phenylpropanoid metabolic pathway (*FaC4H* and *Fa4CL*) had relatively low expression in both ripe HJ and XB flesh. Furthermore, the expression levels of all the above genes in ripe HJ and XB flesh were detected by RT-qPCR to verify the above results (**Figure 2D** and **Supplementary Information 4**). There was a significant association between the gene expression results obtained from RNA-Seq and RT-qPCR (**Figure 2E**). In general, the difference in *FaUFGT* expression levels was the largest between ripe HJ and XB flesh among the genes involved in anthocyanin biosynthesis. We surmised that the inability to accumulate anthocyanins was attributed to lower expression of some anthocyanin biosynthetic pathway genes and very low expression levels of *FaUFGT* in ripe XB flesh.

### Comparison of flavonoid content between red (HJ) and white (XB) strawberry flesh

**Figure 3:**
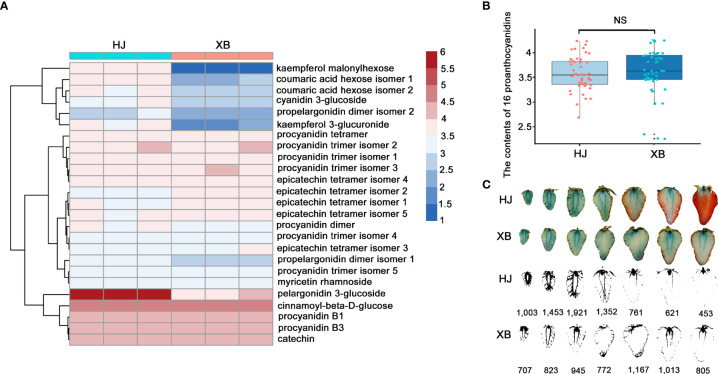
Differences in flavonoid content in red (HJ) and white (XB) strawberry flesh. **(A)** Heat map showing the different contents of flavonoids in red (HJ) and white (XB) strawberry flesh after normalization. **(B)** The content of 16 proanthocyanidins in red (HJ) and white (XB) strawberry flesh. **(C)** Pictures of red (HJ) and white (XB) strawberry flesh dyed with DMAC during fruit development. A darker blue color represents a higher content of proanthocyanidins in strawberry flesh. DMACA intensity was quantified by imageJ software. Significant differences were determined using independent t-tests. "ns" represented no significant difference.

To further verify the very low expression levels of *FaUFGT* accounting for the inability to accumulate anthocyanins in XB strawberry flesh, we compared the flavonoid content between red (HJ) and white (XB) strawberry flesh by UPLC-QTOFMS with an ACQUITY diode array detector. twenty-five primary flavonoids were identified in strawberry flesh, namely sixteen proanthocyanidins, three phenolic acids and glycosides, two flavonol glycosides, two flavonols, and two anthocyanins (**Figure 3A**, **Supplementary Information 5**). The results showed that anthocyanins (pelargonidin 3-glucoside) and proanthocyanidins (proanthocyanidin B1 and proanthocyanidin B3) were the two main flavonoids in strawberry fruits. Pelargonidin 3-glucoside was significantly higher (more than 60 times) in red (HJ) flesh than white (XB) flesh (**Figure 3A** and **Supplementary Information 5**). All the phenolic acids, phenolic acid glycosides and flavonol glycosides were exhibited slightly higher in red (HJ) flesh than white (XB) flesh. However, the contents of majority proanthocyanidins were even slightly lower in red (HJ) flesh than white (XB) flesh, and exhibited no distinct difference in HJ and XB flesh as a whole (**Figures 3A, B**). Several detection methods of proanthocyanidins have been developed based on the reaction of 4-dimethylaminocinnamaldehyde (DMACA)-specific integration with flavan-3-ols (Payne et al., 2010; Dooren et al., 2018). The DMACA reactions showed that proanthocyanidins were primarily produced in fruit at early developmental stages, and there were no significant differences among the contents of total proanthocyanidins in either HJ or XB flesh during fruit development (**Figure 3C**).

### Manipulation of *FaUFGT* expression alters the color of strawberry flesh

**Figure 4:**
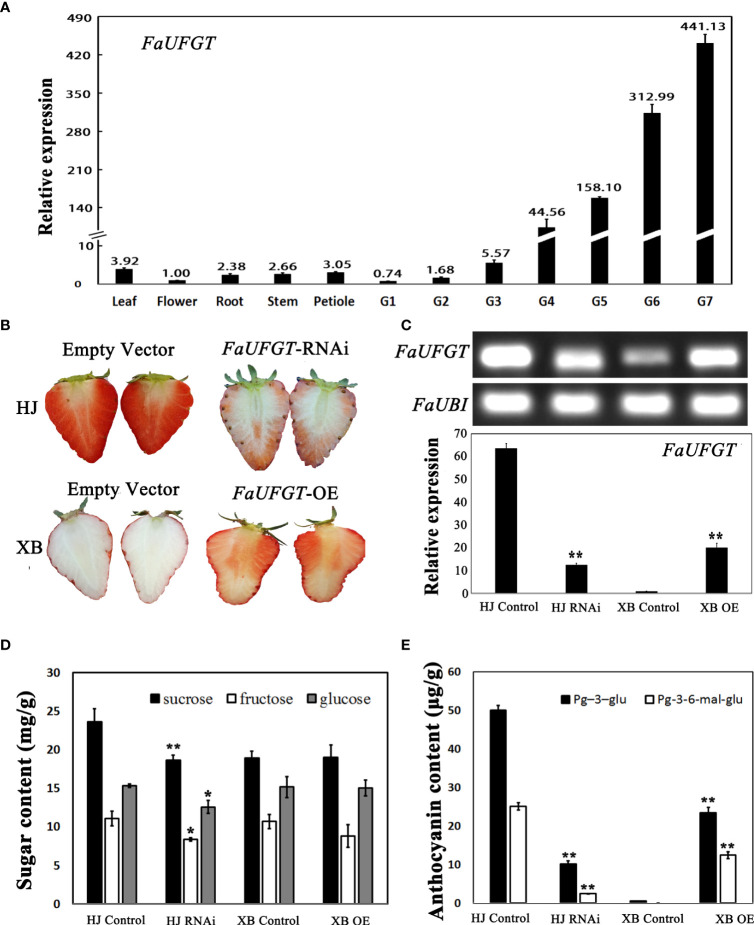
Function of *FaUFGT* in flesh coloration. **(A)** The expression levels of *FaUFGT* detected by RT-qPCR in different tissues of HJ strawberry. Values are the mean (± SD) of three technical replicates. **(B)** Effect of *FaUFGT-RNAi (*HJ*)* and *FaUFGT-OE* (XB) on strawberry flesh color. The top line indicated that when the whole flesh of the control fruits (HJ) completely turned red, the flesh of *FaUFGT*-RNAi (HJ) was pink or red. The bottom line shows that the flesh color of control fruits (XB) transfected with empty vector was still white in the ripe stage, while the flesh of *FaUFGT-OE* (XB) turned red. **(C)** The expression levels of *FaUFGT* in HJ control, HJ RNAi, XB control and XB OE examined by SqRT-PCR and RT-qPCR analysis. **(D)** The sugar content in HJ control, HJ RNAi, XB control and XB OE examined by HPLC. **(E)** The anthocyanin content in HJ control, HJ RNAi, XB control and XB OE examined by HPLC. Asterisks represent significant differences *P < 0.05 and **P < 0.01 as determined using independent t-tests.

The results described above showed that *FaUFGT* was differentially expressed in red and white strawberry flesh but was also expressed in other strawberry tissues. Next, we measured the expression of this gene in the leaves, flowers, roots, stems, petioles and seven stages of flesh of HJ strawberry. Expression analysis showed that *FaUFGT* was primarily expressed in ripe HJ flesh (more than 400 times higher than in other tissues) and was only slightly expressed in other tissues (**Figure 4A**). The expression level of *FaUFGT* rapidly increased in HJ flesh during fruit development, which indicated the important role of *FaUFGT* in anthocyanin accumulation in strawberry flesh.

To determine whether the low expression level of *FaUFGT* (maker-Fvb7-4-augustus-gene-3.55) caused the inability of anthocyanin to accumulate in ripe XB flesh, we used transient overexpression (OE) and RNA interference (RNAi) techniques (Liao et al., 2018). As shown in **Figure 4B**, *FaUFGT*-RNAi clearly inhibited anthocyanin accumulation in HJ flesh. When the whole flesh of the control fruits completely turned red, the flesh of *FaUFGT*-RNAi was pink or red. In contrast, *FaUFGT-*OE successfully turned the white XB flesh back into a red color. To examine whether the phenotypic change in flesh color was indeed correlated with the expression level of *FaUFGT*, we measured it in *FaUFGT*-RNAi and *FaUFGT-*OE flesh. *FaUFGT*-RNAi resulted in a substantial reduction in *FaUFGT* in HJ flesh, whereas *FaUFGT-*OE led to an over 20-fold increase in *FaUFGT* in XB flesh, which indicated that RNAi techniques successfully suppressed *FaUFGT* expression in strawberry flesh (**Figure 4C**; **Supplementary Information 6**). Consistent with flesh phenotypic changes, manipulation of *FaUFGT* expression strongly altered the contents of anthocyanins. Compared with the corresponding control, the main anthocyanin substances (Pg-3-glu and Pg-3-6-mal-glu) significantly decreased and increased by inhibition and overexpression of *FaUFGT*, respectively, while the changes in sugar content were indistinct as anthocyanin content increased (**Figures 4D, E**).

### Identification of candidate SNPs and indels responsible for coloration loss in strawberry flesh

**Figure 5:**
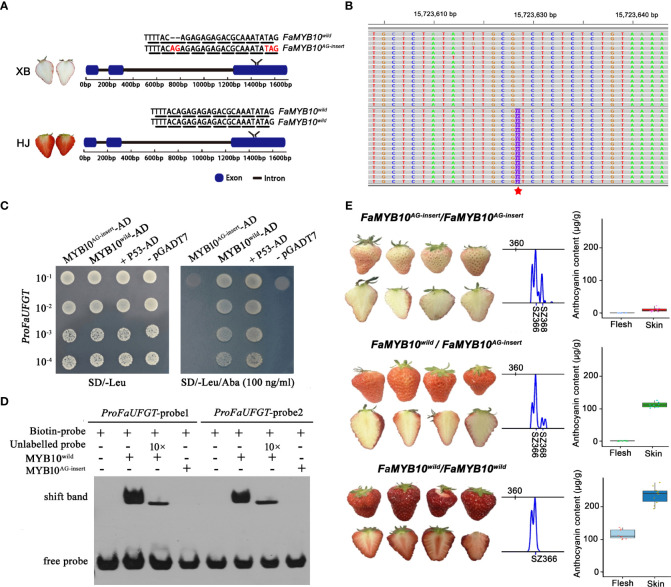
Identification of the mutant gene responsible for strawberry flesh color. **(A)** Diagram showing the genotype of *FaMYB10* in HJ and XB. The chromosome length is indicated at the top. The black triangle indicates the synonymous mutation, the red triangle indicates the nonsynonymous mutation, the red diamond indicates the nonframeshift deletion mutation, and the red five-pointed star indicates the frameshift insertion mutation SNP in *FaMYB10* that causes a premature stop codon. A frameshift insertion mutation (AG insert) presented to one allele of the *FaMYB10* gene in XB. **(B)** Alignment of *FaMYB10* reads from XB ripe flesh transcriptomes to the *Fragaria* x *ananassa* Camarosa Genome by the genome browser IGV. The red five-pointed star indicates the frameshift insertion mutation (AG insert). **(C)** Yeast one-hybrid assays examined the ability of FaMYB10^wild^ and FaMYB10^AG-insert^ to bind the promoter of the *FaUFGT* gene. **(D)** Electrophoretic mobility shift assay confirming the binding of FaMYB10^wild^ to the Myb-binding sites within the promoter of the *FaUFGT* gene, while the FaMYB10^AG-insert^ lost its binding ability. **(E)** The *FaMYB10* genotype, fruit color and anthocyanin content of the selfing progeny of XB strawberry. SZ366 represented that the amplified fragment size was 366 bp without AG-insert. SZ368 represented that the amplified fragment size was 368 bp with AG-insert. There were three *FaMYB10* homoeologs in octoploid strawberry. Only one of them were highly expressed and resulted color formation in the strawberry fruit (Plant Cell. 2020 Dec; 32(12): 3723-3749). So, the three panels represent genotype *FaMYB10^AG-insert^/FaMYB10^AG-insert^*, *FaMYB10^wild^/FaMYB10^AG-insert^*, *FaMYB10^wild^/FaMYB10^wild^* according to left-to-right scanning. The STR primers used are shown in **Supplementary Information 1**: **Table S2**.

The above study indicated that low expression of *FaUFGT* was responsible for the inability to accumulate anthocyanins in ripe XB flesh. However, how transcription factors regulate *FaUFGT* gene transcription in XB flesh has not been fully elucidated. To identify the causative mutation in XB strawberry, we performed whole-genome resequencing of DNA from HJ and XB. In total, 13.67 Gb and 9.17 Gb of 150-bp paired-end reads were obtained for HJ and XB, respectively. A total of 75.02% of reads from HJ and 74.88% of reads from XB were aligned to the strawberry genome (*Fragaria* x *ananassa* Camarosa Genome Assembly v1.0.a1) by BWA mem v0.7.17 (Li, 2013) with default parameters. Next, SNPs and indels were called and filtered, and a total of 1,167,965 variations were identified between HJ and XB samples, including 68,524 SNPs in the coding region (**Supplementary Information 1**: **Figure S1**). From the strawberry genome, we identified 73 genes that encode enzymes of the anthocyanin biosynthesis pathway and R2R3 MYB transcription factors participating in anthocyanin biosynthesis, and a total of sixty-eight variations were identified in the coding regions of these genes in HJ and XB. Forty-five of the variations generated synonymous mutations, nineteen resulted in nonsynonymous mutations, three caused nonframeshift deletion mutations, and only one indel in the exon of marker-Fvb1-2-snap-gene-157.15 or FxaC_2g30690 produced a frameshift insertion mutation (AG-insert) and premature stop codon (**Figure 5B**, represented by a red star in Fvb1-2). Sequence analysis indicated that marker-Fvb1-2-snap-gene-157.15 or FxaC_2g30690 was an *R2R3-MYB* transcription factor (*FaMYB10*) (**Supplementary Information 1**: **Figure S1**). The *F. ×ananassa* cv Camarosa reference genome contains three *FaMYB10* homoeologs with full-length ORF. The mutation was found in the exon of *F. iinumae*-derived subgenome *FaMYB10* (maker-Fvb1-2-snap-gene-157.15 or FxaC_2g30690), which showed dominant expression in the flesh of both ‘HJ’ and ‘XB’ ripe flesh (**Supplementary Information 3**).

We focused on the *FaMYB10*, which was reported to play a dominant role in controlling the pigment accumulation in strawberry peel and flesh (Wang et al., 2020). We investigated the genotype of *FaMYB10* by cloning and sequencing the coding sequence in HJ and XB strawberries. Among twenty clones derived from XB strawberry, the coding sequence of *FaMYB10* in eleven clones was WT (*FaMYB10^wild^*) and could be translated into a complete R2R3 MYB protein (233 amino acid protein), but it contained a frameshift insertion mutation (*FaMYB10^AG-insert^*) in the remaining nine clones, resulting in translation into a predicted shorter protein (154 amino acid protein). The coding sequences of *FaMYB10* in twenty clones from HJ strawberry were all WT and could be translated into a complete R2R3 MYB protein (233 amino acid protein) (**Figure 5A**). Furthermore, we observed that more than 35% of the total *FaMYB10* reads were *FaMYB10^AG-insert^* in XB ripe flesh transcriptomes (**Supplementary Information 1**: **Figure S2** and **Supplementary Information 7**). All the *FaMYB10* reads were determined to be WT in HJ ripe flesh transcriptomes. Next, we examined the ability of FaMYB10^wild^ and FaMYB10^AG-insert^ to bind the promoter of the *FaUFGT* gene. Y1H assays demonstrated that FaMYB10^wild^ was able to bind to the promoter of the *FaUFGT* gene, while the FaMYB10^AG-insert^ could not (**Figure 5C**). An electrophoretic mobility shift assay (EMSA) also confirmed that FaMYB10^wild^ was able to bind to the Myb‐binding sites within the promoter of the *FaUFGT* gene, while the FaMYB10^AG-insert^ lost its binding ability (**Figure 5D**). Then, we analyzed the skin color, flesh color in the selfing progeny (106 single plants) of XB strawberry, and there were three fruit types, deep-red peel red flesh (twenty-four plants), red skin white flesh (fifty-six plants) and pink peel white flesh (twenty-six plants), followed 1:2:1 segregation ratio (**Figure 5E**). We randomly selected ten single plants per fruit type to examined the anthocyanin content and *FaMYB10* genotype. There were three *FaMYB10* genotype, one for each of the three fruit types: deep-red skin red flesh (*FaMYB10* ^wild^/*FaMYB10* ^wild^), red peel white flesh (*FaMYB10* ^wild^/*FaMYB10* ^AG-insert^) and pink skin white flesh (*FaMYB10* ^AG-insert^/*FaMYB10* ^AG-insert^) (**Figure 5E**). There was a significant association among *FaMYB10* genotype and anthocyanin content in flesh and peel (**Figure 5E**).

## Discussion

Previous studies have shown that increases or decreases in strawberry fruit color are generally associated with various natural mutations in *MYB10* transcription factors, such as a 52-bp insertion in the *FvMYB10* coding region (Castillejo et al., 2020) and an ACTTATAC insertion in the *FaMYB10* coding region (Wang et al., 2020). However, the relationship between normal and mutated *MYB10* in the regulation of anthocyanin synthesis gene expression has not been studied to date. In this paper, we demonstrate that heterozygous frameshift insertion mutations (*FaMYB10^AG-insert^/FaMYB10^wild^*) in *FaMYB10* cause a low expression level of *FaUFGT*, which is responsible for the loss of color in strawberry flesh. The peel and flesh color were tightly linked to the *FaMYB10* genotype in the selfing progeny of white-fleshed strawberry. Therefore, the *FaMYB10^AG-insert^* represents a good candidate gene for the molecular breeding of white strawberry.

### Content difference in flavonoids in red (HJ) and white (XB) strawberry flesh

The composition and content of flavonoids determine strawberry quality, which not only determines fruit attractiveness but also contributes to fruit nutritional quality (Mezzetti, 2013; Schaart et al., 2013). This paper makes an overall comparison of flavonoid content between ripe HJ and XB flesh. Pelargonidin and cyanidin are the major pigments that, in the form of pelargonidin-3-glucoside and cyanidin-3-glucoside, account for the bright and dark red color, respectively, in cultivated strawberry (Giampieri et al., 2012). Consistent with the fruit color phenotype, only a small amount of anthocyanin was measured in the ripe flesh of XB, which was in contrast to the high abundance of anthocyanin observed in the ripe flesh of HJ (**Figure 1C**, **3A**). However, there were high levels of proanthocyanidins during whole fruit development in both HJ and XB flesh (**Figure 3**). The content difference in flavonoids in HJ and XB flesh indicated that the anthocyanin pathway was blocked, while the proanthocyanidin pathway was not blocked in white (XB) strawberry flesh.

### Essential role played by *FaUFGT* in the biosynthesis of anthocyanin in strawberry flesh

The anthocyanin biosynthetic pathway has been well studied in many species and involves at least nine crucial gene families that encode enzymes of the anthocyanin biosynthesis pathway. We compared the expression levels of these key genes in the ripe flesh of HJ and XB (**Figure 2** and **Additional file 3**). The majority of these key genes had high expression levels in both HJ and XB ripe flesh in early genes (*FaPAL*, *FaCHS*, *FaCHI*, and *FaF3H*) and late genes (*FaDFR* and *FaANS*). This result is consistent with the high levels of proanthocyanidins, suggesting that the proanthocyanidin biosynthetic pathway is unobstructed in both HJ and XB flesh. Meanwhile, the higher expression level of these key genes is also consistent with the higher content of flavonoids in HJ than in XB ripe flesh. However, *FaUFGT* was clearly differentially expressed (upregulated more than twenty-fold), suggesting that it contributed to the different flesh colors between HJ and XB. Jiang et al. also performed comparative transcriptomic analysis of HJ and XB ripe fleshed, and suggested that the repression of* FaC4H* was responsible for lack of anthocyanin in XB ripe flesh (Jiang et al., 2022). *FaC4H* is a key gene in the phenylpropanoid metabolic pathway. It showed dramatically lower expression in ripe XB flesh than in ripe HJ flesh, which is in agreement with phenolic acids and phenolic acid glycosides were exhibited lower in ripe XB flesh than in ripe HJ flesh (**Figures 2**, **3A**). However, *FaC4H* had relatively low expression in both ripe HJ and XB flesh compared with anthocyanin biosynthesis genes (*FaCHS*, *FaF3H, FaDFR*, *FaANS* and *FaUFGT*), which indicated it was not the most critical gene in anthocyanin biosynthesis in strawberry (**Figure 2**). Moreover, *FaUFGT* was expressed mainly in ripe fruit and little in leaf, flower, root, stem, petiole and immature fruit (**Figure 4A**). This result is consistent with the content distribution of anthocyanin, suggesting the important role of *FaUFGT* in anthocyanin accumulation. UFGT, the last key gene in the anthocyanin biosynthesis pathway, acts as the committed step toward either stable anthocyanin or proanthocyanidin (Griesser et al., 2008). The low expression level of *FaUFGT* may be the main reason for the accumulation of low levels of anthocyanins but abundant proanthocyanidin in ripe XB flesh (**Figure 3**). Lin et al., 2018 performed comparative transcriptomic analysis of red- and white-fleshed strawberry cultivars in two ripening stages, and believed that the downregulated lncRNAs might participate in anthocyanin regulation by acting as targets for microRNAs (miRNAs). To confirm our hypothesis, the gene function of *FaUFGT* was verified*. FaUFGT*-RNAi clearly caused a different phenotype with pink or less red flesh compared to the bright red flesh of the controls (**Figure 4B**). Strawberry injected with agrobacterium carrying the *pBI-FaCHSi* construct could even exhibit completely white fruits in the fully ripe stage (Hoffmann et al., 2006). However, *FaUFGT*-RNAi did not cause the flesh to become completely white in all injected strawberry fruits. Although the expression level of *FaUFGT* was substantially reduced by *FaUFGT*-RNAi, it was still considerably higher (more than five-fold) than that in XB flesh (**Figure 4C**). Therefore, we suspect that this phenomenon is due to the part of the gene-specific sequence chosen for the cloning of the construct, which results in an inefficiency of *FaUFGT*-RNAi. On the other hand, *FaUFGT-*OE successfully turned the white XB flesh back into a red color, but it was less red than the HJ flesh (**Figure 4B**). Meanwhile, *FaUFGT-*OE resulted in a more than twenty-fold increase in *FaUFGT* in XB flesh, but it was still lower than that in HJ flesh.

### Heterozygous frameshift mutation in *FaMYB10*, leading to coloration loss in white (XB) strawberry flesh

A number of studies have demonstrated that gene expression is regulated by promoter sequence variations (Kobayashi et al., 2004; Espley et al., 2007; Zhang et al., 2020) and specific transcription factors (Cutanda-Perez et al., 2009; Schaart et al., 2013; Jin et al., 2016). The promoter and coding sequence of *FaUFGT* were cloned and sequenced, and no difference was detected between HJ and XB, indicating that specific transcription factors may contribute to the differential expression of *FaUFGT* between HJ and XB. R23-MYB transcription factors have been identified as master regulators in the biosynthesis of anthocyanin in strawberry (Aharoni et al., 2001; Castillejo et al., 2020). Notably, the expression levels of *FaMYB10* and *FaMYB1* were not different or were slightly higher in HJ than in ripe XB flesh (Additional file 3, Additional file 4). In *F. vesca*, a gypsy retrotransposon insertion in *FvMYB10* was observed to cause a truncated protein and white fruited phenotype (Castillejo et al., 2020). In the white *F* x *ananassa* strawberry variety Snow Princess, an ACTTATAC insertion in the *FaMYB10* coding region resulted in a predicted truncated protein and loss of the ability to regulate anthocyanin biosynthesis (Wang et al., 2020). We investigated the genotype of *FaMYB10* by cloning and sequencing the coding sequence in HJ and XB strawberries, and it showed that the genotype of *FaMYB10* was heterozygous frameshift mutation (*FaMYB10^AG-insert^/FaMYB10^wild^*) in XB. *FaMYB10^AG-insert^* contained a frameshift insertion mutation resulting in translation into a predicted shorter protein (154 amino acid protein). It had the two repeats (R2R3) of the structurally conserved DNA binding domain, the conserved motif KPRPR[S/T]F, but not the C-terminal region. Y1H and EMSA assays all demonstrated that FaMYB10^wild^ was able to bind to the promoter of the *FaUFGT* gene, while the FaMYB10^AG insert^ could not (**Figures 5C, D**). It indicated that the C-terminal region was important to transcriptional activation function of FaMYB10. Moreover, the anthocyanin content was tightly linked to the *FaMYB10* genotype in the selfing progeny of XB strawberry (**Figure 5E**). Therefore, we hypothesized that the frameshift insertion mutation (*FaMYB10^AG-insert^*) of *FaMYB10* caused the low expression of *FaUFGT* and low anthocyanin content in ripe XB flesh. *MYB10* is a homozygous mutation in both white *F. vesca* and *F* x *ananassa*.cv. Snow Princess, whereas it is a heterozygous frameshift mutation (*FaMYB10^AG-insert^/FaMYB10^wild^*) in XB. Meanwhile, there was still a high expression level of *FaMYB10^wild^* in ripe XB flesh, which conflicts with the low expression level of *FaUFGT* and low anthocyanin content. In citrus, an *MYB* regulatory gene, *Ruby2*, has two alleles, *CgRuby2^Short^* and *AbRuby2^Full^*, which exist in pummelo and orange, respectively and have opposite functions in activating anthocyanin biosynthesis (Huang et al., 2018). One hypothesis is that *FaMYB10^AG-insert^* maybe has a similar function to *CgRuby2^Short^* in inhibiting anthocyanin biosynthesis*. MYB* alleles with TE insertions in the promoters are expressed more strongly, allowing for red fruit peel (Zhang et al., 2019) and red fruit flesh (Butelli et al., 2012), respectively. Another hypothesis is that higher concentrations of functional *FaMYB10* overcome endogenous inhibitory effects in the flesh to allow pigmentation.

## Materials and methods

### Plant material

Strawberry plants were acquired from the China National Strawberry Germplasm Resource Nursery and planted in a plastic greenhouse in Nanjing, China. The development of strawberry fruit was divided into seven stages: small green (G1), large green (G2), degreen (G3), white (G4), initial red (G5), partial red (G6), and full red (G7) according the fruit weight and color, which were minor adjustment of periods reported in previous researches (Jia et al., 2011; Yuan et al., 2019). The flesh and peel of *F.* x *ananassa* fruits were separated by a scalpel, immediately frozen in liquid nitrogen and stored at -80°C.

### Measurement of sugar and anthocyanin contents by HPLC

The strawberry tissue was ground into powder in liquid nitrogen and weighed, and 0.5 g fine powder was placed in 1 ml extracting solution (anthocyanins: 1% hydrochloric acid-methanol solution (v:v); flavonoids: methanol), and extracted by ultrasonication in the dark for 40 min. After centrifugation at 1,2000 rpm for 10 min, the supernatant was passed through a 0.22-µm filter for further HPLC analysis. The anthocyanin compounds were analyzed on an Agilent HPLC-1260 system and separated using a ZORBAX SB-C18 column (5 μm, 4.6 × 250 mm), with mobile phases consisting of 0.5% formic acid (A) and methanol (B). The gradient of phase B was as follows: 0-5 min 100-90%, 5-10 min 90-75%, 10-15 min 75-65%, 15-20 min 65-50%, 20-25 min 50-70%, 25-30 min 70-100%, flow rate of 1.0 mL/min and UV detection wavelength of 520 nm. Standards of cyanidin-3-glucoside and pelargonidin-3-glucoside were obtained from Sigma-Aldrich China (Shanghai). All the samples were measured in triplicate in three independent biological replicates.

The sugar content in strawberry fruit was measured using HPLC (Jia et al., 2011). Standards of sucrose glucose and fructose were acquired from Yuanye Bio-Technology (Shanghai, China). All the samples were measured in triplicate in three independent biological replicates.

### Identification of flavonoid content in strawberry

Flavonoid compounds were analyzed on a Shimadzu LC-20A HPLC system coupled with a hybrid quadrupole TOF TripleTOF™ 5600 mass spectrometer (AB SCIEX). The flavonoid compounds were separated using a Waters ACQUITY UPLC HSS T3 C18 column (1.8 µm, 2.1 ×100 mm), with mobile phases consisting of 0.04% acetic acid (A) and acetonitrile (B) at a flow rate of 0.40 mL/min, and the gradient was as follows: 0-12 min 95-95% B and 12-15 min 95-5% B. The column temperature was 40°C, and the injection volume was 2 μL. The mass spectrometer (TripleTOF™ 5600) was equipped with an electrospray ionization (ESI) interface and operated in positive ion mode over the mass range m/z 200-1500 with a 0.25 s accumulation time. The experiments were repeated independently twice for the identification of flavonoids and three times for proanthocyanidins.

### RNA-Seq

Total RNA was extracted from ripe flesh of two cultivars (Xiaobai and Benihoppe) using a quick RNA isolation kit (Tiangen Biotech, Beijing, China). Libraries were constructed using a NEBNext^®^ Ultra™ RNA Library Prep Kit (New England Biolabs, Beverly, MA, USA) and sequenced on an Illumina HiSeq™ 2500. Raw reads were filtered to exclude low-quality reads and adaptors. Clean reads were mapped to *Fragaria* x *ananassa* Camarosa Genome by hisat2 (Kim et al., 2019). The expression levels of anthocyanin biosynthesis pathway genes and R2R3-MYB genes in each RNA-seq library were measured using the TPM method. Differentially expressed genes (DEGs) was obtained by DESeq2 through TPM normalization, and padj < 0.05 was selected as the threshold.

### RT-qPCR analysis

Total RNA was isolated using the polysaccharide and polyphenolics-rich RNAprep Pure Kit (Tiangen); genomic DNA contamination was eliminated using DNase I (TaKaRa Bio, Shiga, Japan). cDNA was synthesized using an M-MLV Reverse Transcriptase Kit (Promega, Madison, WI, USA). RT-qPCR analysis was performed on the CFX Connect Real-Time PCR System (Bio-Red, CA, USA) using UltraSYBR Mixture (Low ROX) (CWBIO, Beijing, China). The relative gene expression level was measured using the comparative 2^−ΔΔCT^ method against the strawberry ubiquitin gene (Livak and Schmittgen, 2001). Each fruit sample was quantified in triplicate in independent biological replicates and three technical replicates. The primers used are shown in **Supplementary Information 1**: **Table S2**.

### Isolation of promoter and coding sequence of *FaUFGT*

The promoter (1,945 bp from the initiation codon) and coding sequence (1,398 bp) of *FaUFGT* were obtained from the ripened flesh of the strawberry variety ‘HJ’ by gene-specific primers. The whole cDNA sequence of *FaUFGT* was added to **Supplementary Information 1**: **Table S4** (GenBank: MZ442640.1). The promoter sequence of *FaUFGT* was added to **Supplementary Information 1**: **Table S5**.

The primer sequences used for promoter and coding sequence cloning are shown in **Supplementary Information 1**: **Table S2**.

### Transient gene expression in strawberry fruit

Full-length *FaUFGT* cDNA was obtained from the ripened flesh of the strawberry variety HJ. For RNAi, a 559-bp cDNA fragment (from a span of base pairs 354-912) of *FaUFGT* was cloned and inserted into pTRV2. For overexpression, full-length *FaUFGT* cDNA (1,398 bp) was cloned and inserted into pCAMBIA1305. All recombinant plasmids were transformed into *Agrobacterium tumefaciens* GV1301. Agrobacterium infiltration in strawberry was performed as described previously (Hoffmann et al., 2006). Each single colony was inoculated and grown into 10 ml of liquid LB with Kan (50 mg/ml) and Rif (50 mg/ml) medium until the OD600 reached approximately 0.8-1.0. After centrifugation, the supernatant was discarded, and the cells were resuspended in agroinfiltration buffer (10 mmol/L MgCl_2_, 10 mmol/L MES, 20 μmol/L acetosyringone) to reach an OD600 of exactly 1.0. For RNAi, each *Agrobacterium tumefaciens* strain containing pTRV1 and pTRV2 or pTRV2-*FaUFGT* was mixed at a ratio of 1:1, and HJ flesh was injected at the white (G4) stage with a 1-ml needleless syringe. For overexpression, the *Agrobacterium tumefaciens* strain containing pCAMBIA1305-*FaUFGT* was injected into XB flesh at the white (G4) stage with a 1-ml needleless syringe. Fruits were injected with the *Agrobacterium tumefaciens* strain containing empty vector at the same stage as the control.

The primer sequences used for plasmid construction are shown in **Supplementary Information 1**: **Table S2**.

### Identification of genes participating in anthocyanin synthesis

The anthocyanin biosynthesis genes and the R2R3 MYB transcription factors were identified from the strawberry genome by the BLASTP program (Altschul, 1990) using *Fragaria vesca* protein sequences as queries with an E-value < 10^-5^. The candidate proteins were then compared against the NR database to confirm by BLAST.

### Genome read mapping and variant calling between HJ and XB strawberry varieties

Genomic DNA was individually extracted from the young leaves of two strawberry varieties, HJ and XB, and sequenced using the BGI-500 platform. The paired-end reads were processed to remove adapters and low-quality reads based on FASTP v0.20.0 (Chen et al., 2018). The purified reads were then aligned to the recently published strawberry genome with BWA mem v0.7.17 (Li, 2013) with default parameters. Only uniquely aligned reads were used to detect genomic variants with GATK v 4.1.0.0 (McKenna et al., 2010). Variant calling was performed with parameters ‘QD < 2.0 || DP < 5 || MQ < 40.0 || FS > 60.0 || SOR > 3.0’. Small indels were defined as indels with sizes less than 10 bp. Genetic variations between two strawberry varieties, HJ and XB, were extracted and subsequently annotated with ANNOVAR (Wang et al., 2010).

### Yeast one-hybrid

The promoter of *FaUFGT* was amplified and cloned into the pAbAi vector. Next, the recombinant plasmid of pAbAi-bait was linearized and transformed into yeast strain Y1HGold. The subsequent yeast strains were plated onto SD/-Ura selective medium. *FaMYB10^wild^* and *FaMYB10^AG-insert^* were amplified and cloned into the pGADT7 vector to generate AD-*MYB10^normal^* and AD-*MYB10^AG-insert^* plasmids, which were individually transformed into strain Y1H Gold harboring pAbAi-bait and subsequently transferred onto SD/-Ura/AbA medium for further screening. All the primer sequences employed in this analysis are shown in **Supplementary Information 1**: **Table S2**.

### EMSAs

The promoter fragments of *FaUFGT* containing two Myb-binding sites were directly synthesized as biotin end-labeled oligonucleotides by Wuhan Genecreate (Wuhan, China). The biotin end-labeled probes were incubated with the purified recombinant proteins in binding solution (Beyotime, GS005) with or without unlabeled probes at 25°C for 30 min. Next, the samples were detected by 6% polyacrylamide gels. Gel images were captured by a BK-L96C luminometer (Biosino). The primers used are shown in **Supplementary Information 1**: **Table S3**.

## Data availability statement

The datasets presented in this study can be found in online repositories. The names of the repository/repositories and accession number(s) can be found in the article/**Supplementary Material**.

## Author contributions

HY and MZ conceived and designed the project. HY, WC, XC, and FP performed the experiments. HY, MZ and JW analyzed the data and wrote the paper. All authors contributed to the article and approved the submitted version.

## Funding

This work was supported by National Natural Science Foundation of China (32102336), Jiangsu Academy of Agricultural Science and Technology Innovation Fund project (CX(21)3033) and Jiangsu Province Major Agricultural New Varieties Creation Project (PZCZ201721).

## Conflict of interest

The authors declare that the research was conducted in the absence of any commercial or financial relationships that could be construed as a potential conflict of interest.

## Publisher’s note

All claims expressed in this article are solely those of the authors and do not necessarily represent those of their affiliated organizations, or those of the publisher, the editors and the reviewers. Any product that may be evaluated in this article, or claim that may be made by its manufacturer, is not guaranteed or endorsed by the publisher.
