# Supplementary material for: Heterozygous frameshift mutation in FaMYB10 is responsible for the natural formation of red and white-fleshed strawberry (Fragaria x ananassa Duch)
Source: Front Plant Sci. 2022 Oct 26;13:1027567. doi: 10.3389/fpls.2022.1027567 (PMC9644031; doi:10.3389/fpls.2022.1027567)
Supplement: Supplementary file 1 [file Table_1.docx]

Table S1 HPLC-MS/MS analysis of anthocyanins in strawberry fruit

| Peak | Retention Time (min) | MS (m/z) | MS/MS (m/z) | Anthocyanin |
| --- | --- | --- | --- | --- |
| 1 | 12.90 | 611 | 449/287 | cyanidin-3,5-di-O-glucoside |
| 2 | 15.50 | 449 | 287 | cyanidin-3-glucoside* |
| 3 | 16.90 | 433 | 271 | pelargonidin-3-glucoside* |
| 4 | 17.60 | 463 | 301/286 | peonidin-3-glucoside |
| 5 | 19.10 | 535 | 287 | cyaniding-3-O-(6-O-malonyl-b-D-glucoside) |
| 6 | 20.30 | 519 | 271 | pelargonidin-3-O-(6-O-malonyl-b-D-glucoside) |
| 7 | 21.90 | 533 | 271 | pelargonidin-3-O-methyl-(6-O-malonyl-b-D-glucoside) |
| 8 | 22.20 | 563 | 301、286 | peonidin-3-O-methyl-(6-O-malonyl-b-D-glucoside) |

* Confirmed with reference standards


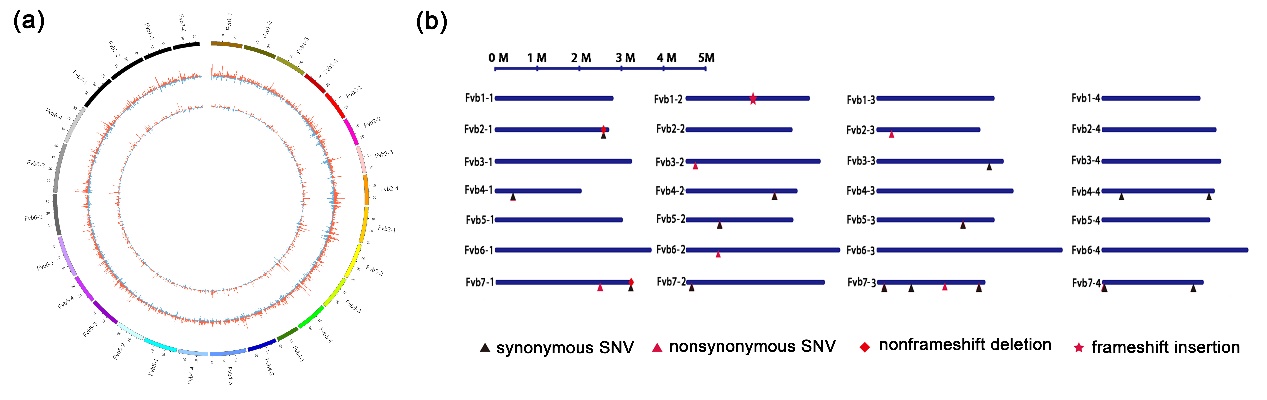


Figure S1. Identification of the mutant gene responsible for strawberry flesh color. (a) Genome-wide distribution of SNPs and indels between HJ and XB samples. (b) Diagram showing the locations of high-quality exonic SNPs between the HJ and XB genomes. The chromosome length is indicated at the top. The black triangle indicates the synonymous mutation, the red triangle indicates the nonsynonymous mutation, the red diamond indicates the nonframeshift deletion mutation, and the red five-pointed star indicates the frameshift insertion mutation SNP in *FaMYB10* that causes a premature stop codon. A frameshift insertion mutation (AG insert) presented to one allele of the *FaMYB10* gene in XB.


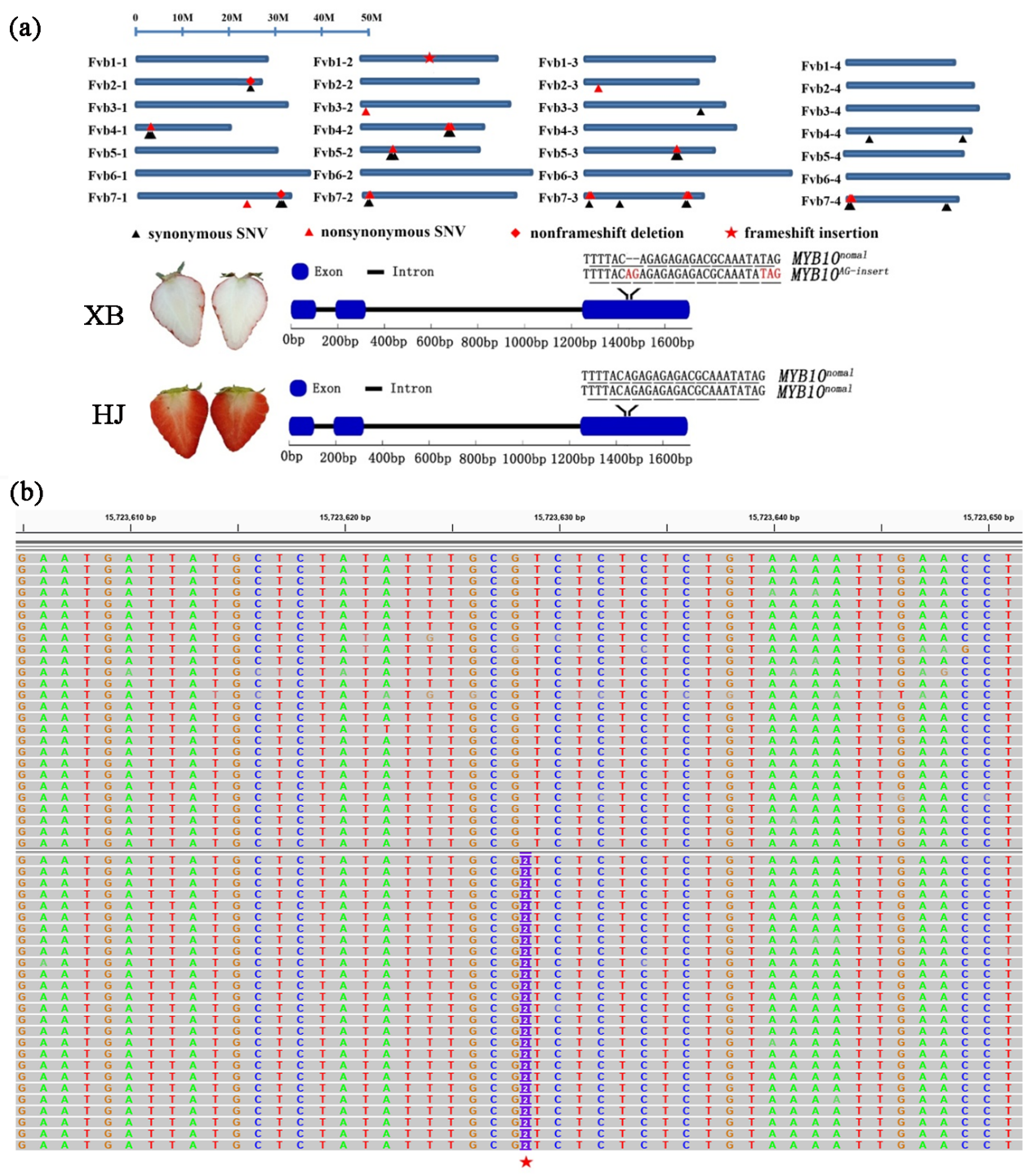


Figure S2. Alignment of *FaMYB10* reads from XB ripe flesh transcriptomes to the *Fragaria x ananassa* Camarosa Genome by the genome browser IGV. The red five-pointed star indicates the frameshift insertion mutation (AG insert).

Table S2 The sequences of the oligonucleotide primers used in this work.

| Primer Name | Primer Sequence (5'→3') | Experiment |
| --- | --- | --- |
| *FaPAL1* | F:CAGTGACCAACCATGTCCAG R: TCCTCCAAATGCCTCAAATC | qRT-PCR |
| *FaPAL2* | F:GCTGAGCAACACAACCAAGA R: GAGATCAACAGCTTGGCACA | qRT-PCR |
| *FaC4H* | F:GACGGTTCCTTTCTTCACCA R:CCTCGCTTTCAAATCTCCTG | qRT-PCR |
| *Fa4CL1* | F:GGTGCTGAAGAGTGGAGGAG R:CGATCTTCAATTCGGCATTT | qRT-PCR |
| *Fa4CL2* | F:CTCGTGTGATAAGGATGTGTTGC R:GGTTTTCACCGTCGACCTGC | qRT-PCR |
| *Fa4CL3* | F:TTCAGGTGGTCACCATCGAC R:GCCACACTTGTGATGAGACTCTTG | qRT-PCR |
| *FaCHS* | F:GCCTGAGAAGTTAGAAGCCACG R:CGAACCCAAACAGAACACCC | qRT-PCR |
| *FaCHI* | F:AGCATCACCCTCTACCCTCAT R:ACCCACTGCACCCATAGCTG | qRT-PCR |
| *FaF3H* | F:TGTGGCGTTTGAGTCCGAGA R:TGACGAGCTGATGGGGTTGG | qRT-PCR |
| *FaF3`H* | F:TCCTTGACTCGCTGCCTTGT R:CGTTCGGTGATGAAGCTCGA | qRT-PCR |
| *FaDFR1* | F:CAGGGTTTGAGTTCAAGTACAGC R:TATCGCCATTCTCCTGCTTCT | qRT-PCR |
| *FaDFR2* | F:AGGGCATTGAGGAGAACTTGAC R:GCATGAACCACTCGCTAACCA | qRT-PCR |
| *FaANS* | F:ATCTTCTCCTTGGGCGGCTC R:AACATGGTTCCCGGTCTGCA | qRT-PCR |
| *FaUFGT* | F:CAAGCAATCCAACAGCTCAATC R:GAAAACATACCCCTCCGGCAC | qRT-PCR |
| *FaANR* | F:ATAGGTTCAACTCCGACCGC R:CCCGTTGAAGGTCCGAATGA | qRT-PCR |
| *FaFLS* | F:AGCTCCAATATCTTGCCCTCCA R:AGTAGTCAAGCTGTGTGGGAGA | qRT-PCR |
| *FaLAR* | F:GTGAGGCGTGCGATTGAGA R:TCGACGAAGTAGGCTTTAACC | qRT-PCR |
| *FaMYB1* | F:CCAAATAAGCCCCATGAGAA R:TCAACTCAGGCACCAAACAG | qRT-PCR |
| *FaMYB10* | F:GAGAAGGCAAATGGCATCAT R:TTCATCCTCTGCAAACTCTCC | qRT-PCR |
| *FaUBI* | F:CAGACCAGCAGAGGCTTATCTT R:TTCTGGATATTGTAGTCTGCTAGGG | qRT-PCR |
| *Gene-FaUFGT* | F:CCAGATACGTAGCAGCTGAGG R:GCAGAAATGTGTTTCTTGCTTGTC | Gene cloning |
| *Pro-FaUFGT* | F:TCGCAAACACCAAGATGACAC R:CACCCACCTGGTTTGATAGTAC | Promoter cloning |
| *pCAMBIA1305-FaUFGT* | F:CGGGATCCATGGCACCAGTATCAAACCAG R:GCTCTAGAATTGGTTGTAGCCATTTCCAACAA | Transient gene expression |
| *TRV2-FaUFGT* | F:GCTCTAGACTTGGTCACTGACGCCTTCT R:CGGGATCCTAGCGCCATAAGCTCTTCCG | Transient gene expression |
| *pAbAi-FaUFGTpro* | F:AAAAGCTTGAATTCGAGCTCGGTACCTCGCAAACACCAAGATGACAC R:TATACATACAGAGCACATGCCTCGAGCACCCACCTGGTTTGATAGTAC | Yeast one-hybrid |
| *FaMYB10^normal^-AD* | F:ACGACGTACCAGATTACGCTCATATGGGGGGTTTCGGTGTGAGAAAAG R:TATCTACGATTCATCTGCAGCTCGAGTCACACGTAGGAGATGTTGAC | Yeast two-hybrid |
| *FaMYB10^AG-insert^-AD* | F:ACGACGTACCAGATTACGCTCATATGGGGGGTTTCGGTGTGAGAAAAG R:TATCTACGATTCATCTGCAGCTCGAGCTATATTTGCGTCTCTCTCTC | Yeast two-hybrid |
| *FaWD40-BD* | F:TGATCTCAGAGGAGGACCTGCATATGGAGAATTCGACCCTCGAATC R:TGCGGCCGCTGCAGGTCGACGGATCCTCAAACCTTCAAGAGCTGCATC | Yeast two-hybrid |
| *STR-FaMYB10* | F:TGTAAAACGACGGCCAGTAAAGAACTGCCAATGATGTGAAGAAC R:CATGATCCTCTAAACCAAGACCAG | Capillary electrophoresis |

Table S3 The whole cDNA sequence of of *FaUFGT*

CCAGATACGTAGCAGCTGAGGTCTAAGTATTTACGTACGTAGCTTGATCACTAGCTAGAAATGGCACCAGTATCAAACCAGGTGGGTGGTCATGTGGCGGTGCTAGCCTTTCCCTTCTCCACTCACGCCGCCCCGCTGTTGAACATCGTGTGCCGCCTCGCCGCCGCCGCGCCAAGCACTCTCTTCTCTTTCTTCAACACCAAGCAATCCAACAGCTCAATCCTAGCAGGCAACACGTCCGTACTGCGTTACAGTAACGTAAGTGTGTGTGAGGTGGCGGATGGTGTGCCGGAGGGGTATGTTTTCGTGGGTAAGCCACAGGAGGACATAGAGTTGTTCATGAAGGCTGCCCCGGACAACTTCAGGAGGTGCTTAGAGGCGTCGGTGGCGGAGTCCGGGAGGGAGGTCAGCTGCTTGGTCACTGACGCCTTCTTTTGGTTCGGTGCTCATATGGCGGATGACATGGGAGGAGTGCCGTGGGTGCCGTTCTGGACCGCCGGACCGGCTTCACTCTCGGCTCATGTACACACTGATCTCATCAGGAACACAACTGGTGGGGGTGGTCACGATGAGAAGGAAACCATCACTGTCATTGCAGGAATGTCGAAAGTGAGACCTCAGGATCTGCCAGAGGGAATCATCTTCGGAAACTTGGAGTCGCTCTTTTCACGTATGCTTCACCAGATGGGACAGATGCTACCACTTGCAACCGCAGTTTTCATCAACTCCTTCGAAGAACTAGATCCTGTGATCACAAATGATTTGAAGTCCAAATTCAAGAGGTTCCTCAACGTGAGACCATTGGACCTACTAGAACCACCAGCAAGTGCAGCCACCACCACACCGCAGACGGCGGAAGCTGTTGCCGGAGATGGCTGCTTATCGTGGCTTGACAAACAGAAGACGGCGTCCGTGGTCTATGTGAGTTTTGGATCAGTAACAAGACCATCGCCGGAAGAGCTTATGGCGCTAGCTGAGGCTCTGGAGGCTAGTAGGGTTCCATTCTTGTGGTCACTTCGGGACAACTTAAAGAACCCACAGCTAGATGAGTTCCTAAGCAAAGGAAAGTTGAACGGAATGGTGGTGCCTTGGGCGCCACAACCACAGGTCCTGGCGCATGGTTCAGTTGGAGCCTTTGTAACACATTGCGGTTGGAACTCGGTGCTTGAGAGCGTAGCAGGTGGAGTGCCTTTGATTTGCAGGCCTTTCTTCGGCGATCAGAAACTTAACGCGAGGATGGTAGAGGATGTGTGGAAGATTGGTCTCAGGTTGGAGGGTGGGGTTTTCACCAAGAATGGCATGCTTAAGAGTTTGGACATGCTATTATCACAAGACGAGGGGACCAAAATGAAAAACAAGATCAACACACTAAAACAACTCGCACAACAGGCCGTAGAACCAAAAGGGAGCTCCACTAGGAACTTTGAGTCATTGTTGGAAATGACCACAACCAATTAAGCACAACTACAACTACATTCCACAAGAATAAAGAGTTTCTGAGAGTTACAGTCGATTGAACAAGAGAGCTACATTTTAGACAGATTTAACTGTGGAGAAAAAAACACCAAGCATTGAGGCAACGATATGAATAAGACAAGCAAGAAACACATTTCTGCAATCTCTAGAGG

Table S4 The promoter sequence of *FaUFGT*

TCGCAAACACCAAGATGACACTGTTGAGCATAAATGGATCACTGATAATGGCCTGAAGAAGAAAACACACATAAGTGAGAAACAATAAGTGGCCAATGTATCTGAACTGTATTTCTTTCTGGACATGATACCTGAAATTCTTCCTTGGCTCGTCCAATTCTCTCTCTGTCCAAGGAGTCAACAACATAAATCTGCATAGGCAAAAGTACGTATATCAAGAAAATTGTGAGTATTGAAGCAATAACAAAGCCAAAAGATGTAGACTAGGATACGAGAAAACACAAAAGTAATGAAAAGAGACACAATTGACTAGCAGGAATCATAATGCAGTAGAACAAAACAGTACCAGTCCATCCGTATTATTAAAGTAATGCCTCCATAGTGGCCTTAGTTTCTCCTGCCCACCAACATCCCAAACTGTGAACATCACATTCTTGTACTGAACTTTTTCCACATTAAAACCTGTTCATAAAAAGGGAACCTCAGTATGCCGACAACAAAATCAACAGATATATACCGTCTACCCATTGGGTGTGGTTATATAATAGCCACTTTGAACATGCCCGATTGCTTATACTTCTCAGATTAAAGAGTCTCTTGATATAGAAAACATGTACACATGCTAACATTATGACAGTCAAATAACAACTTCCCAACAACAGAGCCTCATGTATCTAGCCGTTGAAAATACCAACCTATTGTAGGAACAGTTGACAAGACTTCTCCAATGTGCAGTTTGTAAAGGATGGTTGTTTTACCAGCCGCATCTAGTCCAAGCATTACAACCTAAGCCACCACAAACAGAAATGATAATTGATATAAGGAAAGTGGATCAAAGGTGATCCGAGTAAAACACATGCTTCTTTTTAAATCTTCTTTGTGTTCTCTACTACATTCGGCTACGAAAATAGGATAAGTATTTGTTGTGTAAACCAGCTTATATATTTATCGGAATTAATGTGATCATACAAGAATCTCATCATCTTCATCATCATCATCATCGTCATTCTAATTTGAGGAACATATTTGCAGCAATTTCACCATCACCATCCATAATTAGAAGAAAAATGAACCAAAAGATGAGTAAATTCACACAGAAGATACCCGATACGAATATTCGCAATACGATTCAAACTACTTAGAAGAAGAATTGAGAATTTCAACTAAATTGAACCTAAAACAAAACAATTAATCACTTCTTTCTATAAACCCTAGAACTCCCTGGGCAAGTATGATCATCAATCAAAACAAGAAAAATGGCAAAAAAAAAATCGTACGAAATTAAAAAATGAAAATTCAAAGAGAAAGGGAAAGAGTGAGTACTCGCATCTCAGTATTGCCGAAGAAGGCATCGAAGAGCTTGCGAAAAGCTTGACCCATGGCGACGAAACTTCAACACCAAAAAACAAAAGCAGAGAGGAAAAGAAAACGTCGTCGATCTCTCTTTCTCTCTTTCTCTGTGTAAAGTATCGACGAACTATATGAGCTGGGCGACTAAATTAGCAGAGCTGCGGTGGCGATCGATTTTTAGAGAGAGAAAGCCAGCCACGATGAGATTTTAGTGAGAGAAAATAGAAACACAAAGGTCGCGTTCTATTCTAATTTCTCTGCACCCCAAAAAAGAAGAAAGATCTTTCATTTTCTACTATTGGGTCTATTGGGCTTATTCTTGAAATGGGCCACTGATCTCAAAACAATTTAGCCCATGGGCCTGCCCGGGGGTTGTCGCGTGCTTCGAACCCTACGGTCTCCTGAAGAGTGGTTGTTGGATTGTGACAACCACCATTAGTGTAACAGTGACTGCTGTATAAATGGAGAGCAATGCCTATAAATGGAGAGCAATGCACATGTACAAAATTGGTAGAGGAATCACATTCACTTGAGTAGGTCCAGATACGTAGCAGCTGAGGTCTAAGTAGTTACGTACGCAGCTTGATCACTAGCTAGAAATGGCACCAGTACTATCAAACCAGGTGGGTG
